# Mexican Scientist Diaspora in North America: A Perspective on Collaborations With México

**DOI:** 10.3389/frma.2022.898896

**Published:** 2022-06-02

**Authors:** Paulina Gómez-Flores, Vicente Morales-Salgado, Angélica Maza, Aline Villarreal, Linda R. Lara-Jacobo, Mónica Ivette Jiménez-Córdova, Daniel Jiménez-Alvarez, Alma Cristal Hernández-Mondragón

**Affiliations:** ^1^Asociación Mexicana para el Avance de la Ciencia, Ciudad de México, México; ^2^Departamento de Biotecnología, Universidad Autónoma Metropolitana Unidad Iztapalapa, Ciudad de México, México; ^3^Centro de Estudios de Derecho e Investigaciones Parlamentarias, Ciudad de México, México; ^4^Facultad de Economía, Universidad Nacional Autónoma de México, Ciudad de México, México; ^5^Departamento de Fisicoquímica, Facultad de Química, Universidad Nacional Autónoma de México, Ciudad de México, México; ^6^Faculty of Science, The University of Ontario Institute of Technology, Oshawa, ON, Canada; ^7^Secretaría de Investigación y Posgrado, Instituto Politécnico Nacional, Mexico City, Mexico; ^8^Program on Science, Technology and Society, Center for Research and Advanced Studies of the National Polytechnic Institute, México City, México

**Keywords:** scientific diasporas, brain drain, Mexican scientists, United States, Canada, international collaborations

## Abstract

Scientific diasporas from developing countries represent an opportunity to strengthen international collaborations. These collaborations build upon the desire of members of the diasporas to establish scientific, academic, technological, and cultural exchange networks with the communities in their country of origin. While Mexico has a significant number of scientists residing abroad, particularly in North America, and most of them are committed to aid in the country's development, institutional coordination has not harnessed its benefits. In this work, we present an analysis of initiatives carried out by Mexican scientists, members of the diaspora, studying or working in the United States of America and Canada. The study is based on a set of interviews with members of this diaspora. We asked scientists about the conditions that enabled or obstructed their initiatives back in Mexico, and we discussed the role of these factors for capacity building. We also provide general recommendations to enhance contributions to the advancement of science in the country.

## Introduction

Knowledge has become the main input for economies that aim to increase productivity and global competitiveness (Castells, 2000). Indeed, in this so-called Knowledge Economy, scientists play a key role (World Bank., 2013). Thus, in terms of the investment made by a country to possess a qualified labour force, emigration represents a loss of human capital that undermines opportunities for its development. In this regard, the concept of brain drain has been introduced as “the permanent emigration of skilled persons from one jurisdiction to another” (Tigau, 2009, p. 49) or, in terms of qualification, it is defined as “the output of those who have at least a university degree, with at least 15 years of schooling, in areas of science or technology and who work in those fields” (Martuscelli and Martínez Leyva, 2007, p. 4). Indeed, brain drain is usually regarded as a problem for developing countries, like Mexico, since it implies the reduction of a scarce type of workforce: people with hard-gained know-how.

Although brain drain implies a loss of skilled labour, it can also become an agent of international cooperation and development (Tejada, 2012; Jarquín-Solís, 2022). This can be key for developing countries with an output of scientists moving abroad since their collaborations with public administrators, business communities, and research organisations have shown to support robust innovation environments (UNCTAD., 2017, p. 17). In this context, scientific diasporas have been defined as “self-organised communities of expatriate scientists and engineers working to develop their home country or region, mainly in science, technology, and education” (Barré et al., 2003).

Scientific diasporas consider people that “have been integrated into the labour, social and cultural dynamics of the country where they live, but who are willing to collaborate in the establishment of scientific, academic, technological and cultural exchange networks with the communities of their place of origin” (Martuscelli and Martínez Leyva, 2007). Scientific diasporas can play an important role in leveraging migration's benefits because they carry opportunities for international collaborations to promote knowledge networks and capacity building for the countries involved. For example, diasporas from Colombia, India, and South Africa have shown successful results in proposing public policies, training human resources in science and technology, and, in general, building capacities for the development of their country of origin (Martuscelli and Martínez Leyva, 2007).

The case of the Mexican diasporas is of particular importance. Mexico is the second country with the highest emigrant population in the world, and the first place in Latin America (McAuliffe and Triandafyllidou, 2021). As of 2020, around 11 million people had emigrated from Mexico most of them to the United States of America (USA) (McAuliffe and Triandafyllidou, 2021). It is estimated that in the coming years, Mexican emigration will continue to increase, although not in a sustained manner (Secretaría de Gobernación., 2022). According to the Mexican National Council of Science and Technology (CONACyT), between 1990 and 2015 around 1.2 million Mexicans with graduate and postgraduate degrees left in search of better opportunities abroad (OECD, 2020 cited in Rogozinski, 2020). This is a significant number considering that around 80 percent of the Mexican population does not hold a university degree (OECD, 2020 cited in Rogozinski, 2020). The main “receiver” of these migrants is the USA, where around 80% of all Mexicans abroad live (McAuliffe and Triandafyllidou, 2021). Gaspar and Chávez (2016) reported that 13.4% of all Mexicans with postgraduate degrees in the USA perform in the field of basic sciences.

In Mexico, few initiatives have benefited from the Mexican diaspora, and fewer have exploited it. One of them, which includes the USA and Canada, is the Red Global MX (Global MX Network), formerly the Red de Talentos Mexicanos en el Exterior (Network of Mexican Talents Abroad), a governmental institution established in 2006 through joint work between the Institute of Mexicans Abroad of the Ministry of Foreign Affairs (or Instituto de los Mexicanos en el Exterior-IME), the Ministry of Economy, and the CONACyT. The Global Mx Network brings together individuals interested in promoting the development of Mexico, with a particular focus on inserting Mexico into the knowledge economy. It is organised in local chapters, autonomous in terms of management and action (Gaspar and Chávez, 2016).

As of 2022, the Global MX Network is made up of 71 Chapters in several countries around the world and 19 Nodes in different states of Mexico. It has more than 6,500 active members in 34 countries (RGMX Capítulo Países Bajos, 2020). A Global Coordination of the Network connects the performance of the chapters, although there are also four Regional Coordinations: Canada, USA-Latin America, Europe, and Asia-Oceania, which are responsible for assisting the organisation of the Chapters regionally and representing the network before national and foreign institutions, as well as serving as interlocutors with the IME (RGMX Capítulo Países Bajos, 2020; RGMX Capítulo Portugal, 2022).

Each Chapter and Node procures its own resources. They possess autonomy of management and action to participate in national and international initiatives. They also have elected Board of Directors, and define their own rules, work plans and objectives. Nonetheless, it is expected that they work in four strategic sectors: (1) Science, Technology, Research and Academia; (2) Entrepreneurship and Innovation; (3) Social Responsibility; and 4) Creative Industries (RGMX Capítulo Países Bajos, 2020; RGMX Capítulo Portugal, 2022). Some Chapters have, in addition to undertaking projects in these sectors, other programs to support the quality of life of Mexicans abroad and to promote Mexican culture (RGMX Capítulo Países Bajos, 2020).

Another initiative around the Mexican diaspora in North America is the Confederation of Mexican Graduate Students and Researchers in Canada, a non-governmental organisation established in 2006, whose motto is “Creating community networks of specialists for the promotion of culture and academic exchange between Mexico and Canada”. Its general objectives are: (1) To guide Mexican students, researchers, and professors, (2) To create a network of contacts that includes students, researchers, government, and industry, in order to facilitate academic and employment opportunities both in Canada and in Mexico, and (3) To inform and support the academic community, as well as cultural and altruistic activities that allow transmitting Mexican culture (CEIMEXCAN, 2014).

The opportunities posed by the movement of scientists, whether in natural or social sciences, as well as humanities conducting empirical research, from México to the USA and Canada, is precisely the topic of this article. This paper examines collaboration experiences of members of the Mexican scientific diaspora in North America. We aim to understand the role of the scientific diaspora in leveraging knowledge networks and building multinational science, technology, and innovation environments that promote development in Mexico. We analyse the importance of enablers, challenges, and barriers that scientists encountered when establishing scientific collaborations.

The article is organised as follows: in Methods and data we describe the methods and data collected and used for the study, namely an initial questionnaire and a set of interviews with members of the Mexican scientific diaspora; in Findings we present the results of the analysis focusing on identifying enablers or obstructors of collaborations between members of the diaspora and initiatives in Mexico; in Discussion we discuss the results, and give some final remarks.

## Methods and Data

In this research, members of the Mexican scientific diaspora are considered people who recognise themselves as Mexicans and possess postgraduate studies in science and technology, whether natural or social, as well as humanities conducting empirical research, and who are currently living in the United States or Canada. This work is indeed a perspective of the mobility of qualified Mexican human resources in the northern region of the continent and the opportunities for multinational collaborations. Thus, this qualitative analysis commences with an initial questionnaire that collects demographic data on the Mexican scientific diaspora in North America and continues with interviews with those who accepted the invitation to participate in them.

The sample presents the difficulty of having no exhaustive database that gathers Mexican scientists in North America. However, there are several networks where members can join at will, given that each network successfully reaches its target population. The networks used to reach members of the diaspora were both formal (Delegación general de Québec en México) and informal, as well as social networks (Twitter, Facebook, and LinkedIn). As a result, 29 scientists responded to the initial questionnaire, and nine of them were interviewed.

**Figure 1:**
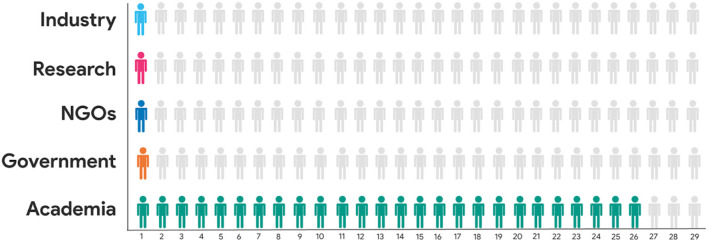
Sectoral distribution of respondents of the initial questionnaire.

**Figure 2:**
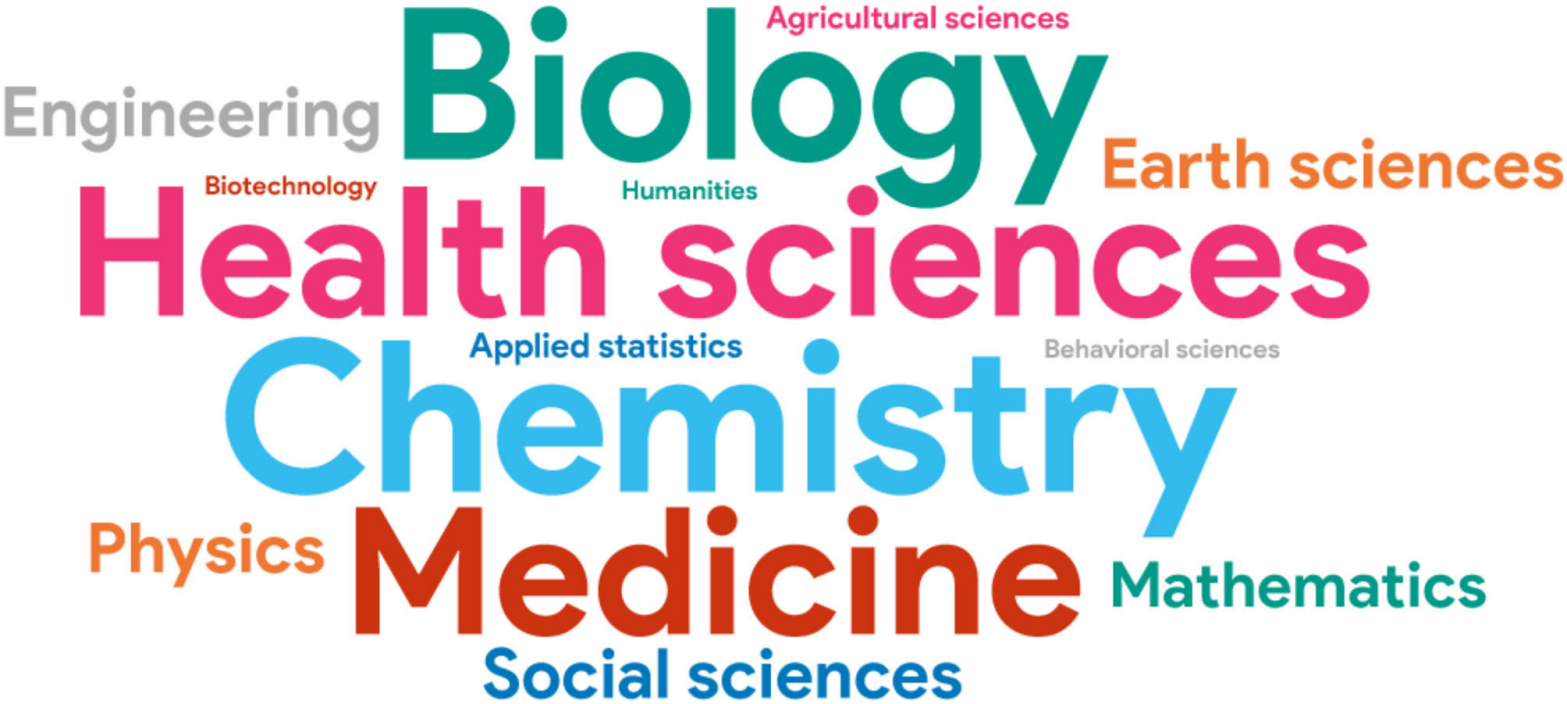
Area of specialisation of respondents of the initial questionnaire.

The median age of respondents was 35 years; 61% identified themselves as female, 36% as males, and 3% as non-binary. Regarding the country of residence, 71% reside in the USA and 29% in Canada. The median time of residence of the respondents is 5.7 years in their corresponding country. Most of the scientists that answered the questionnaire hold a postdoctoral researcher position, but other types of research positions, as well as postgraduate students also participated in the initial survey. One of the respondents performs as a consultant and as a postdoctoral researcher. Most respondents perform within the academic sector (see Figure 1), and most of them do so in Medicine, Health Sciences, Biology, and Chemistry (see Figure 2). Note that the respondents were able to select more than one option as an answer for these questions.

In the next stage of data collection, interviews were conducted with respondents that agreed to comment further on their situation as members of the diaspora. The interviews were based on open questions, which made it possible to delve into the reasons for migration, as well as opinions on the obstacles and enablers of collaborations with stakeholders in Mexico. The guiding questions for the interviews were the following:

- Could you describe your professional training?
- Could you describe the two main reasons that determined your decision to move to your current residency?
- Could you describe your collaboration attempts with Mexican initiatives?
- According to your experience, what do you consider to be the main challenges in establishing successful collaborations with Mexican initiatives?
- In your opinion, what do you consider enables collaborations with Mexican initiatives?
- Do you consider it helpful to have an institution dedicated to permanently lead, manage, and implement collaborations between Mexican scientists abroad and initiatives in Mexico? If so, how do you envision such an entity's functionality?
- Do you have any additional comments, observations, or suggestions?

All interviews were recorded, and data was collected. However, this information is not published for protection of personal data.

Five of the nine interviewees identify themselves as women and four as men. Their time residing outside of Mexico ranges from 2.5 to 7.5 years. While three of them live in Canada, the remaining six live in the USA. Eight interviewees hold a Ph.D. and work in postdoctoral positions, and only one is still in graduate school. Two scientists work in medicine and health sciences; two in biology, chemistry, medicine, and health sciences, two in social sciences; one in biology, chemistry, and engineering; one in social sciences and applied statistics; and one in physics, mathematics, and earth sciences.

## Findings

The initial questionnaire allowed us to survey the attempts of the diaspora to collaborate back with Mexican initiatives, as well as the sense of need for additional mechanisms that facilitate such efforts, before performing the interviews. When asked about the attempts, successful or not, 77% of participants stated that they have tried to collaborate, while the rest admitted not having tried. Regarding the need for mechanisms other than existing ones, the position was somewhat divided, with 48% stating the sufficiency of existing mechanisms, 40% expressing the need for more mechanisms, and 12% admitting not being sure.

Nine respondents of the initial questionnaire agreed to be further interviewed. In what follows, we will describe the answers received during these interviews. It is worth mentioning that the interviews were performed in Spanish. We show the number of responses corresponding to each category in parenthesis.

First, when asked about the reasons to move outside of Mexico, participants commented the following as their main reasons: search for academic growth (4), lack of opportunities for specialisation (3), lack of professional working options (2), and lastly, personal reasons (1).

Regarding attempts to collaborate with Mexican initiatives, it is noteworthy that participants preferred collaborations with Mexican institutions in the form of academic projects (8). Other answers included projects with non-governmental organisations (NGOs) (2), with governments (2), and with industry (1). Participants mainly expect to produce high-impact scientific publications and to participate in conferences on their area of specialisation as products of their collaboration.

Participants were also asked about the main challenges they faced while establishing collaborations with Mexican initiatives to elaborate on what hinders cooperation. Respondents identified a lack of knowledge of the available mechanisms, mismatch of timelines between academic and non-academic activities, and difficulties in setting common goals and objectives as the main obstacles to successful collaborations.

According to the experience of one of our interviewees:

“… academic collaborations between Mexico and the USA are easier because there is a common goal: conducting research. In the case of NGOs there are many areas of opportunity and opportunities for synergy, considering the experience of both parties. In this case, the most complicated is establishing common goals and schedules, because timing in NGOs and/or the government are rather different, maybe they need short-term results, while research results take longer.”

The interviewees also pointed out that in most cases, these reasons are enough to result in a loss of interest by one of the parties, leading to the cease of the project. Other obstacles noted include the inefficiency of bureaucracy in both Mexican, and American or Canadian institutions, lack of funding and economic incentives, poor soft skills, and difficulty in locating collaborators, mainly due to missing institutions dedicated to supporting initiatives of Mexicans abroad. Some participants mentioned that bureaucracy hinders the formalisation of collaborations agreements and affects the success of the ones previously established.

When we look at enablers of collaborations, according to the experiences of most interviewees (8), previously established professional and personal networks are of primary importance. Scientists who lacked these networks found it hard to advance their partnerships. For example, one interviewee stated:

“… the collaborations where I have participated, have occurred because collaborators are my friends, and because they are mainly acquaintances from my master's or bachelor's studies, that is how the relationships are built. In the case of people who have contacted me after giving me an internship, it is worth noting that most of them come from abroad, from other Latin American countries, for example, Colombia, but they have never contacted me from Mexico except when there are previous relationships…”

Another respondent who has worked directly with the Mexican government at the national and state levels said:

”... the complicated thing is to have a point of contact to establish a communication channel, I think I had the advantage and the fortune of having worked in the federal public administration... and my job had great exposure to people who are in key positions. That allowed me to have certain points of contact when I went to study for a doctorate. It was a privilege to have met those people because otherwise I would have to knock on doors.“

Finally, participants were asked about the necessity for a third-party liaising with Mexican scientists in North America and stakeholders in Mexico. Most interviewees agreed that an NGO (7) would be more suitable than a government office (1) due to the need for more direct and close cooperation. In addition, the interviewees consider that lasting collaborations and agreements are more fruitful if these do not depend on political circumstances, mainly because of a deficient follow-up by Mexican administrations. In the words of one of our respondents:

“... coming from the government there is the advantage of funding, but I consider that, independently of who is in the government at any given time, someone is always left out. On the other hand, coming from the civil society as an NGO there is the advantage of inclusion because there are no other interests but the ones from the own society...”

Remarkably, participants were eager to collaborate back with Mexico, mainly in the areas of the advancement of science and the promotion of scientific opportunities abroad. It is also worth mentioning that scientists reached through the survey belong to a specific subset of the Mexican scientific diaspora in North America: early-career scientists from certain knowledge areas who have rather recently left Mexico (no more than 10 years), mostly seeking better working conditions and opportunities in the USA and Canada.

Scientists surveyed showed interest in maintaining ties with Mexico through international collaborations. They seek to obtain personal and professional growth; to develop a multinational science, technology, and innovation environment; and harness science results for societal benefit in Mexico, Canada, and the USA.

## Discussion

Cooperation between nations is essential in some areas, whether it be economic, cultural, environmental, or academic (Johnson, 2015; Jorge-Pastrana et al., 2018). Even more, diasporas can be part of a new form of governing through responsabilisation of their welfare and that of their communities (Kunz, 2010). Scientific diasporas can be key agents in starting, growing, and sustaining cooperation in the form of concrete initiatives (Marmolejo-Leyva et al., 2015; Valenzuela-Moreno, 2021). This becomes particularly relevant when diasporas come from developing countries like Mexico, where its members could be regarded as promoters of collaborations rather than just part of the brain drain. This study explored the views of 29 members of the Mexican scientific diaspora, who work or study in the USA and Canada, providing a description of their initiatives and an analysis of the challenges and barriers encountered by them.

Although migration has been studied from different perspectives, this work focuses on the attempts of emigrants to establish collaborations back with Mexico. It is generally considered that brain drain occurs because satisfactory conditions are not found in the country of origin and/or that conditions in the country of destination are regarded as better (Giannoccolo, 2009). Answers from the scientists interviewed confirm this observation.

Respondents stated that there is significant interest in the Mexican scientific diaspora to collaborate with Mexico, mostly within their area of expertise. When attempts have been made to collaborate, previously established personal networks were commonly used. Thus, the interviews also confirmed the dichotomous situation of emigrants, who feel committed to the country that has welcomed them but maintain strong ties of identity with their country of birth (Martuscelli and Martínez Leyva, 2007).

We observed that academic collaborations are the most frequent type of collaboration carried out by respondents. This situation can be due to the fact that the current assessment of scientists in Mexico prioritises academic production in the form of research articles over other activities (CONACyT, 2020). The type of collaborations reported by interviewees is in line with what is expected for Latin American countries, such as increasing mobility opportunities for students, publishing papers with colleagues in their country of origin, participating in science communication activities, and linking knowledge with private, public, and non-governmental organisations (Bonilla, 2022).

There is also an outspoken interest from respondents to help increase the training of new human resources and improve opportunities for young Mexican scientists abroad. However, commitment from a few enthusiastic and knowledgeable, yet isolated, agents is not enough. Support in the form of coherent, coordinated, and collaborative institutional commitments, as well as relevant stakeholders' involvement have shown to be of grave importance (Tettey, 2016).

It is also noticeable that respondents declared that there was no institutional follow-up of successful collaborations between Mexican and foreign institutions, neither from Mexico nor from institutions abroad. This translates into difficult conditions for coalitions to promote effective collaboration and build sustainable community change (Goodman et al., 1998). Scientists interviewed identified an NGO as a desirable option to help avoid drawbacks of government initiatives, such as lack of continuity, impersonality, bureaucracy, and even unwillingness. Such an option implies the self-organisation of a multinational scientific community to aid Mexican administrations that, so far, have not successfully coordinated the efforts of the country's scientific diaspora.

The respondents also recognise a lack of so-called soft skills as an obstacle that affects the commencement of collaborations; agreement of goals, timelines, and responsibilities; and the way the team deals with administrative requirements. Indeed, soft skills are key since collaborative capacity is highly influenced by existing skills and knowledge, the attitudes that members bring, and the efforts made to build, support, and access this capacity (Foster-Fishman et al., 2001).

It is remarkable that more formal mechanisms to communicate and collaborate, such as permanent exchange programs (Kramer and Zent, 2019), specific financing for collaborative projects (Science Fund of Republic of Serbia SFRS., 2019), a scientific diaspora networking or a platform registration (European Commission, 2022), were not mentioned by the respondents. These mechanisms can be developed to support institutions focused to engage and improve Mexican scientific diaspora's initiatives, e.g., (Bravo and De Moya, 2018; Martínez-Schuldt, 2020; Sánchez and Cantú, 2021). As we have seen in the introduction, some formal mechanisms exist, but neither these nor other ones were mentioned by the interviewees.

Even though a significant number of Mexican scientists reside in the United States or Canada, few studies present updated and reliable data about this diaspora. This could be due to the methodological, organisational, and financial challenges of this type of studies. As a contribution to better understanding the opportunities posed by the Mexican scientific diaspora, we have examined the experiences and attitudes of some of its members towards building collaborations with their country of origin. This study invited members of the diaspora by means of formal and informal channels. Yet, a low level of response was registered in both the initial survey (29 participants) and the ensuing interviews (9 participants). Further efforts to reduce drawbacks and efficiently direct initiatives that connect Mexican scientists abroad are needed, particularly in North America.

While the Mexican scientific diaspora in North America exists, it is not yet fully studied and understood as a societal and technological phenomenon that can harness its importance in the dynamics between the countries. To take advantage of the opportunities of possessing a valuable scientific diaspora, Mexico can implement mechanisms that identify, connect, and cohere emigrated scientists towards the common goal of contributing to its scientific and technological development. These mechanisms can profit from connecting members of the diaspora and the scientists residing in Mexico, as well as strengthening existing ones. Given that promoting science and technology in the best possible manner is the goal, we believe this could greatly enhance collaborations and advance science, notably in countries where it is most needed.

## Data Availability Statement

The raw data supporting the conclusions of this article will be made available by the authors, without undue reservation.

## Author Contributions

All authors listed have made a substantial, direct, and intellectual contribution to the work and approved it for publication.

## Conflict of Interest

The authors declare that the research was conducted in the absence of any commercial or financial relationships that could be construed as a potential conflict of interest.

## Publisher's Note

All claims expressed in this article are solely those of the authors and do not necessarily represent those of their affiliated organizations, or those of the publisher, the editors and the reviewers. Any product that may be evaluated in this article, or claim that may be made by its manufacturer, is not guaranteed or endorsed by the publisher.
